# Learning From Agility, Partnership and Innovation During the Covid-19 Pandemic: A Perspective From Industry

**DOI:** 10.3389/fcimb.2022.838565

**Published:** 2022-02-17

**Authors:** Ana María Cárdenas, Celine Roger-Dalbert

**Affiliations:** Becton, Dickinson and Company, Life Sciences – Integrated Diagnostic Solutions, Sparks, MD, United States

**Keywords:** COVID-19, SARS-CoV-2, medtech industry, healthcare industry, diagnostics

## Abstract

Two years after the COVID-19 pandemic started, the world continues to adapt to the profound effects that Severe Acute Respiratory Syndrome Coronavirus 2 (SARS-CoV-2) has had on our lives. As the global crisis took hold, many looked to the medical technology/device industry for guidance and solutions. All while the industry itself, was disrupting its own processes and activities. In order to evolve and deliver accelerated innovation the industry had to be agile, resilient and collaborative with the broader healthcare community and technology partners. Now comes a time when we will start to see what changes were temporary and which ones will become part of the new process, but one thing is certain, we will not be going back to where we were pre-pandemic.

## Agility

As the COVID-19 pandemic disrupted labor markets, the medical technology (medtech) industry had to adapt its practices. Accommodations needed to be made for associates working from home and new considerations were necessary in order to support associates, mainly in manufacturing and research and development, who needed to be at work physically. Adapting meant implementing worksite restrictions to reduce the spread of the novel coronavirus (United Sates Department of Labor and Occupational Safety and Health Administration (OSHA), 2021), as well as personnel screening, testing, and vaccination to reduce both the individual risk and community risk and limit viral spread. Associates required to be on-site to provide customer service were those most severely impacted. Consequently, it was necessary for the industry to adapt and find new ways to support customers remotely. In some instances our instruments offer remote monitoring; allowing the extraction of de-identified databases to support timelier offsite troubleshooting and providing near real-time analytics such as positivity rates or trends in cycle threshold (Ct) values. This is a practice that we should certainly continue to push but that is not always easy to implement because of data security and protection surrounding health information. In addition, we developed tools to replace in-person trainings of customers and associates with virtual offerings. One such example was the creation of virtual environments where customers interact with the solution and associates can train remotely. This agility was especially beneficial when borders closed and travel to customer locations was almost impossible.

Early on during the pandemic, the need to switch gears to rapidly develop and deploy assays to diagnose infection with SARS-CoV-2 lead to a large mobilization of the workforce towards creation of point-of-care and lab-based antigen, antibody and molecular diagnostic assays. In order to support this demand, not only did the labor force have to shift priorities but there was also an increased demand to hire the right talent and expand teams in a short amount of time. Conversely, as fluctuations in hospital procedure volumes and deferral of elective procedures affected other segments of the medtech industry, balancing prevention of layoffs with increasing demand on manufacturing pushed the industry to become more nimble. While there was unpredictable fluctuation in demand and production volumes, the industry had to not only face a shortage in workforce, but also a workforce affected by quarantine measures. Companies took different approaches towards hiring practices and incentives and implemented more flexible work hours to adjust the manufacturing output to the demand. Other functions also stepped up to provide support to the manufacturing function in order to keep up with the increased demand.

Unpredictable fluctuations in manufacturing, supply chains, raw materials and transport logistics of products across the globe led the medtech industry to look for ways of minimizing risk. By diversifying the supply base and validating multiple in-country and external suppliers we can lessen the dependence on high-risk sources and by opting for vertical integration of the supply chain, we can streamline our operations. We have also taken advantage of process innovations such as integration of upstream manufacturing steps or materials for molding of parts. Process improvements and incorporation of new technologies will need to be revisited alongside the trade-off between product variety and capacity flexibility to maintain agility.

## Partnership

In order to accommodate the needs of patient care during the pandemic, key partnerships emerged. Industry partners, manufacturers, and suppliers came together in addition to the public health sector, clinical laboratories, regulatory agencies, non-profit organizations and governments worldwide. The enormous redeployment of resources to ensure rapid regulatory authorization allowed us to develop and launch COVID-19 diagnostic assays in 90 days, compared to what would have normally taken more than 3 years. Institutions fostered unprecedented collaboration between diverse and interdisciplinary groups, allowing for an open exchange of information and rapid learning from one another. The sharing of risk mitigation strategies, efficiencies gained, and innovations may not have happened without the pandemic, and should continue for the long term. If we have learned anything, it is the importance of cross-segment dialog and strong collaboration.

Early on in the pandemic, collection devices were in very short supply. Companies including Copan, Roche, Thermo Fisher, Abbott and BD worked together to evaluate the possibility of validating alternate collection devices as a group, agnostic to the platform used. The group also worked with the United States Food and Drug Administration (FDA) to assess potential options along these lines. We also created partnerships with new swab or collection device suppliers to augment supply and validated the compatibility of their swabs with various molecular or antigen assays. Since different regions of the world had access to different swab suppliers, we tried to ensure availability of solutions across the globe. There was also a lot of lobbying and support from non-profit organizations, like the Bill and Melinda Gates Foundation, to create a network of startup and established companies to foster collaborations and bring to market new diagnostics tools, new technologies or help with manufacturing of new products.

The laser sharp focus on combating a public health emergency negatively affected the approval process of non-COVID products as well, as review processes took longer than anticipated (United States Food and Drug Administration (FDA), 2021). Leaner but still quality driven practices to facilitate approval of medtech products is one of the lessons learned that we hope is here to stay. Based on learnings from COVID-19, new product development best practices that give access to new technologies faster will require collaboration with regulatory bodies as well as post-launch efforts/activities for the medtech industry. Better coordination to prioritize access and sharing of viral genome sequence information, clinical specimens and data, in addition to having commutable international standards and reference materials, would also accelerate the development and validation of diagnostic tools worldwide.

Increased harmonization in the requirements and regulations surrounding the validation and distribution of diagnostic tests would be beneficial. A complex reality is that different countries have different requirements when it comes to study design validations, acceptance criteria and clinical specimen types required. In order to answer specific needs during the COVID-19 pandemic, we found ourselves having to perform additional internal validation studies that added time, effort and increased pressure on an already overloaded workforce. One such example was obtaining claims for saliva testing for France with specific clinical specimen collection for this intended use while claims elsewhere were for nasal swabs. In other instances we collaborated with clinical or public health laboratories or institutions who may have self-validated a given application or published studies utilizing our products. This way we could leverage real-world evidence in order to build the regulatory filing requirements needed to meet local regulations.

## Innovation

Deployment of different kinds of tests that did not need to rely on the already overburdened healthcare systems such as point-of-care testing, and more recently at-home testing, drove the development of added functionality and digital connectivity. Not only have we seen the creation of platforms that allow for remote data capture and instant access to testing results, but also technological innovations facilitating activities that previously required in-person contact. A recent example of this digital innovation is the development of our at-home digital test kit that combines the use of the Veritor rapid test with the use of a smartphone to interpret, deliver and display results without relying on human interpretation.

The at-home test portfolio expansion will continue, driven more by a better understanding of what tests have value to be done in an at-home setting and less on the specific technologies used. Prescribed infectious disease diagnostic tests have significant benefits to public health as they help reduce exposure of staff members and patients and decrease large volumes of individuals seeking care at health care facilities. COVID-19 drove a significant number of hospitals to implement hospital-at-home programs and widespread adoption and successful implementation still hold promise (Balatbat et al., 2021). At-home test portfolio expansion could help lessen the load for overburdened clinical and public health laboratories that struggle with additional testing volumes and staffing shortages. For infectious disease diagnostics, at-home molecular platforms may take hold as they provide increased clinical sensitivity, but antigen assays are less expensive, more available, and continue to be used in instances when obtaining rapid results (15 min or less)has an actionable impact on human behavior, such as when planning a gathering with friends and family.

Both medtech and healthcare industries are at an inflection point where the way health care gets delivered is changing. The use of telecommunications and digital information technologies to access and facilitate health care services remotely, otherwise known as telehealth, is expanding at a rapid rate (Koonin et al., 2020). We have witnessed digital developments when using QR codes readily available for restaurant menus, event platforms to facilitate educational activities, fitness and activity tracking devices and remote health-related patient monitoring. The next generation of technologies need to improve the quality of health care by increasing access to care and helping the clinician be more efficient. For example, expansion of home hospitalizations will move diagnostic testing closer to the at-home patient and transform the healthcare system by affecting clinical sample collection and testing as well as data transmission and clinician utilization of information. This creation of new care settings will foster innovation in the medtech industry. In addition, when looking at at-home testing for chronic diseases, protein quantitation and collection of other specimen sources, such as blood, will be required. Therefore, new technologies allowing for simpler sample collection and transportation while still ensuring sample integrity will expand. Here once again, the need for more efficient and adaptable manufacturing stands out.

## Discussion

The majority of our response to the SARS-CoV-2 pandemic was reactive, and therefore overwhelmed resources all at once. We developed and deployed a plethora of diagnostic assays in an impressively short period, implemented surveillance systems and other technologies to increase connectivity, yet data access remained slow. We still need better mechanisms to track infection, react quicker, and continue to work closely with public agencies so we can reach near real-time data for ongoing and new infections. We will need to continue to close the gap to get information between individual test results and healthcare providers for follow-up and surveillance testing. There needs to be improved coordination between regulatory and development aspects of diagnostic tests. We have significantly increased the speed at which we can roll out tests but still lack the centralized ability to do so, especially in the United States, at a larger scale. The two phase response of 1) having lab developed tests and home brewed diagnostic assays followed by 2) emergency use authorization of low-cost, high-quality commercial assays took longer than what we needed it to be.

We have achieved adequate and reliable direct-to-consumer and decentralized testing without compromising test accuracy or quality and yet there is still a lot of confusion about what types of diagnostic tests to use when. Further partnerships between academia, public health and industry can provide clarity on getting the right test, at the right place, at the right time. Especially when endemic COVID-19 is the likely reality. As we rely more on telehealth, adequate self-collection and guided diagnostic therapy, there needs to be better interactions with healthcare workers and care outside of the hospital setting. In addition, it is necessary to leverage digital technology to promote real-time surveillance and early warning systems if new infections start to emerge in hot spots or specific areas.

A byproduct of this pandemic was a significantly reduced 2020-2021 influenza season. The use of community control tools (masks, social distancing, hand hygiene) to reduce the spread of other pathogens, especially during winter viral season, proved effective. It remains to be seen if we can incorporate behavioral changes that decrease mortality caused by other viral pathogens. Another area that needs attention is how to shift the focus towards prevention and apply lessons learned from quick identification and treatments strategies. For example, an area where we need to leverage surveillance and prevention is towards antimicrobial resistance.

The medtech industry responded to the ever-changing needs posed by the SARS-CoV-2 pandemic and continued to deliver healthcare solutions from bedside care to at-home testing. Recent digital innovations will improve the industry’s ability to continue to support the healthcare community and public health sector in the future. This environment of stronger collaboration and partnerships will facilitate the deployment of new approaches to personalized medicine and diagnostic testing innovations to provide better holistic solutions throughout the patient care continuum.

## Data Availability Statement

The original contributions presented in the study are included in the article/supplementary material. Further inquiries can be directed to the corresponding author.

## Author Contributions

AMC: conceptualization, writing, and review and editing; CR-D: conceptualization, writing, and review and editing. All authors contributed to the article and approved the submitted version.

## Conflict of Interest

AMC and CR-D are current employees of Becton, Dickinson, and Company.

## Publisher’s Note

All claims expressed in this article are solely those of the authors and do not necessarily represent those of their affiliated organizations, or those of the publisher, the editors and the reviewers. Any product that may be evaluated in this article, or claim that may be made by its manufacturer, is not guaranteed or endorsed by the publisher.
